# Expression of mycobacterium tuberculosis induced SOCS3 and STAT3 and the implications on innate immunity in TB patients vs healthy contacts in high TB/HIV endemic setting: A cross-sectional analytical study

**DOI:** 10.1371/journal.pone.0263624

**Published:** 2022-07-15

**Authors:** Patrick Lungu, Kabaso Mushota, Evarist Njelesani, Thomas Sukwa, Shabir Lakhi, Peter Mwaba

**Affiliations:** 1 Department of Internal Medicine, University of Zambia School of Medicine, Lusaka, Zambia; 2 USAID Sustaining Technical and Analytic Resources (STAR) Project, Lusaka, Zambia; 3 Faculty of Medicine, Lusaka Apex Medical University, Lusaka, Zambia; Hamad Medical Corporation, QATAR

## Abstract

**Background:**

Mycobacterium tuberculosis (TB) remains a disease of global health concern and a leading cause of mortality arising from an infectious agent. Protective immunity to TB remains unclear. Suppressor of cytokine signaling-3 (SOCS3) and signal transduction and activator of transcription-3 (STAT3) genes have shown potential to influence innate immunity. We, therefore, explored the expression of SOCS3 and STAT3 and their implications on the innate immunity in TB patients and their healthy close contacts.

**Methods:**

We recruited 72 TB patients and 62 healthy contacts from a high TB and HIV endemic setting (Lusaka, Zambia). We used RT-PCRT and flow cytometry to quantify the expression of SOCS, STAT3 and cytokines respectively. Data was analysed Stata version 14.0 and figures were developed in GraphPad prism version 9.1.0 (221). Assessment for associations for categorical and continuous variables was analysed using the Chi-square test and Mann-Whitney test respectively. Spearman’s rank correlation was used to evaluate the relationship between SOCS3 and IL-6. A *p*-value < 0.05 was considered statistically significant.

**Results:**

Healthy contacts markedly expressed SOCS3 in both unstimulated and stimulated whole blood in comparison to TB patients (*p* <0.0001). STAT3 was elevated in TB patients in TB patients in stimulated blood only. IL-6 (*P* = < 0.0001) and IL-10 (*P* = <0.0001), were significantly expressed in Healthy contacts in comparison to TB patients. TNF-α (*p* = 0.044) were markedly elevated in TB patients in comparison to healthy contacts. IL-6 and SOCS3 correlated significantly in healthy contacts only (r = 0.429, *p* = 0.02).

**Conclusions:**

Both SOCS3 and STAT3 are genes of importance in mounting protective innate immunity against TB. We propose that SOCS3 stimulation and inhibition of STAT3 as possible approaches in gene therapy and vaccine development for TB.

## Introduction

Mycobacterium tuberculosis (TB) remains one of the public health challenges of global concern. TB remains the leading cause of death globally arising from an infectious agent [1]. TB elimination has been absorbed as one of the sustainable development goals to be attained by 2030. There has been momentum built in the response to the TB epidemic to reduce the TB incidence towards elimination. The current declines in the global incidence fall far short of the target. The global decline in TB incidence is about 2% against the target of 4–5% annually [1,2]. One of the key strategies to accelerate the fall in TB burden the development of the TB vaccine. The only TB candidate vaccine currently in use is the Balcille Calmette Guerin (BCG) is now 100 years old and has poor efficacy in preventing TB disease. Its major benefit is in preventing severe forms of TB [3,4].

The World needs a newer TB vaccine that is highly efficacious in preventing TB disease. Several attempts to develop newer candidate vaccine for TB have not yielded a better efficacy than the BCG for indefinite reasons. Understanding the mechanism and factors responsible for resistance to TB may inform approaches to vaccine development and various TB preventive measures. Resistance to TB has been linked to genetic influence. High levels of expression of the suppressor of cytokine signaling (SOCS3) genes (proteins) has been linked to protective immunity in TB [5] SOCS3, together with signal transduction and activator of transcription signaling-3 (STAT3), are the genes that have since been shown to influence the inflammatory response. SOCS3 inhibits of STAT3 by binding to JAK kinase resulting into regulation of cytokine signaling. SOCS3 is essentially a promoter of inflammation. STAT3 a member of the STATS protein family acts as a transcription factor in modulating pro and anti-inflammatory responses. In principle STAT3 promotes an anti-inflammatory response [5,6].

SOCS3 primarily inhibits IL-6 activation of STAT3 [7]. SOCS3 also inhibits the negative effect of IL-6 on the expression of IL-12 in the dendritic cells, which in turn leads to the expression of IFN-γ by the CD4 cells. Interferon gamma (IFN-γ) is central in maintaining the integrity of the ghong focus. IFN-γ restricts the growth of mycobacterium tuberculosis in the macrophages, preventing the establishment of TB infection and progression from latent TB infection to active TB disease [8,9].

An activation of STAT3 leads to an anti-inflammatory response that allows for mycobacterial replication leading to active disease. Masood et al revealed that healthy endemic controls expressed higher levels of SOCS3 when compared to patients with pulmonary TB and those with disseminated TB [10].

Animal studies show that Mouse deficient in SOCS3 has increased susceptibility to TB and is likely to have a lethal form of TB [11]. In this, study we sought to explore the expression of SOCS3 and STAT3 in TB patients in comparison to their healthy contacts when exposed to the wild type mycobacterium tuberculosis.

We hypothesized that healthy contacts of TB patients express a higher level of SOCS3 than the TB patients and that TB patients express higher level of STAT3 compared with their healthy contacts. We, therefore, evaluated the interaction of the two genes and their role in protective immunity against TB.

## Methods

### Study population

The Study population included 72 TB patients at the onset of anti-tuberculous treatment and 52 healthy household contacts. We used a constellation of a negative Genexpert, negative sputum culture and a normal chest roentegraphy to rule out active TB disease in the healthy contacts. Treatment experienced TB patients were excluded and contacts that were found with active TB during the screening were classified as active TB patients. The study included both HIV negative and person living with HIV in both cohorts. Willful written consent was obtained from all study participants.

### Study type

This is a prospective observational study.

### Study settings

The study was conducted at the University Teaching Hospital a national referral hospital in Lusaka, Zambia.

### Ethical approval

Ethical approval was obtained from the University of Zambia Biomedical Research Ethics committee (UNZABREC) and the National Health Research Authority (NHRA). Ethical approval reference number 012-01-18.

## Laboratory procedure

### Cell culture conditions

From each participant we collected 1mL of fresh whole blood into a heparinized bottle and divided into two halves (each containing 0.5mL). One half was unstimulated, and the second half was stimulated with 10 μg/mL of heat killed wild type strain of MTB (H37RV). Whole blood was suspended in glutamine supplemented RPMI 1640 solution. 100 U/ml penicillin-100 μg/ml streptomycin was added to each culture the cultures were conducted at 37°C, with 5% CO2. O.5mLs of supernatants was harvested at 24 hours post-incubation and stored at -70°C.

### Cytokine quantitation

Cytokine expression was measured using the Flow Cytomix human Th1/Th2, IL-17A CBA kit (Bender Med systems GmbH, Vienna, Austria), as per the Manufacturer specification. The detection limit for IFNγ, IL-6, TNF-***α***, IL-4, IL-10, and IL-2, were set as per manufacturer’s instructions. Data output from BD FACS Calibur 4 color flow cytometry was analysed using BD FCAP array software v3.0 no 652099, San Jose, USA).

### Real time PCR

Total RNA was isolated from whole blood both unstimulated and stimulated using Trizol reagent (Invitrogen, USA). RNA (1 μg) was reverse transcribed using MulV reverse transcriptase (Invitrogen, USA) as described previously. Real time PCR was performed in duplicate 20 μl reactions containing SYBR green qPCR ready mix (Sigma-Aldrich), 150 nM forward and reverse primers, for SOCS3 and STAT3. Primers for GAPDH were run at the same time as the primers for the two genes of interest. We used Roche Applied-science LightCycler®480 Ⅱas recommended by the manufacturer of the primers [12]. Human primers SOCS3, STATS3 and GAPDH were acquired from Sino biological. We used the Livak method (Ratio = 2^–ΔΔCT^) for relative quantification of the expression of the total RNA for SOCS3 and STATS3.

### Statistical analysis

The data was double-entered into the questionnaire and Microsoft Excel 2010 (Microsoft Corp., Redmond, Washington, WA, USA), and statistical analysis was performed using STATA statistical software version 14 (STATA Corp, Texas, TX, USA) and GraphPad prism version 9.1.0 (221). Non-parametric continuous variables were expressed as medians and IQR. Assessment for associations for categorical and continuous variables were analysed using Chi-square test and Mann-Whitney test or Kruskal- Wallis respectively. We used Wilcoxon signed–rank test for analysis for significance in non-parametric paired samples. Spearman’s rank correlation was used to analyse the relationship between IL-6 and SOCS3. A *p*-value < 0.05 was considered statistically significant.

## Results

We enrolled 72 TB patients and 524 of their healthy contacts in the study. 51 (70.8%) and 33 (64.5%) were males among TB patients and healthy contacts, respectively. We observed no significant difference between the two cohorts in the distribution of age, gender and residence. We found a significant difference in the HIV seropositivity between the two-study population, 40 (55.6%) TB patients and 10 (19.2%) were persons living with HIV, and all were on antiretroviral treatment. Further, we observed that TB patients in comparison to the healthy contacts had significantly lower CD4 count. Likewise, TB patients had lower body mass index (BMI) when compared to their healthy contacts (Table 1).

**Table 1:** Demographic and clinical characteristics of TB patients and their healthy contacts.

Variable	TB patients n = (72)	Healthy contacts n = (52)	*P* value
**Gender**			
Male	51 (70.8%)	33 (64.5%)	0.386
Age, Median (IQR)	33 (26–38)	35 (27–48)	0.141
**Residence**			
High density	52 (72.2%)	42 (80.8%)	0.273
**HIV status**			
Positive	40 (55.6%)	10 (19.2%)	< 0.0001*
**CD4 Count**(cell/mm^3^), Median (IQR)	452.5 (236–607.5)	723.5 (438.5–872.5)	< 0.0001*
**B**MI (Kg/m^2^), Median (IQR)	20.5 (18.3–22.4)	21.4 (18.8–25.1)	0.048*

Data expressed as: n (%), median (IQR) ^*^Denotes statistical significance.

**Fig 1:**
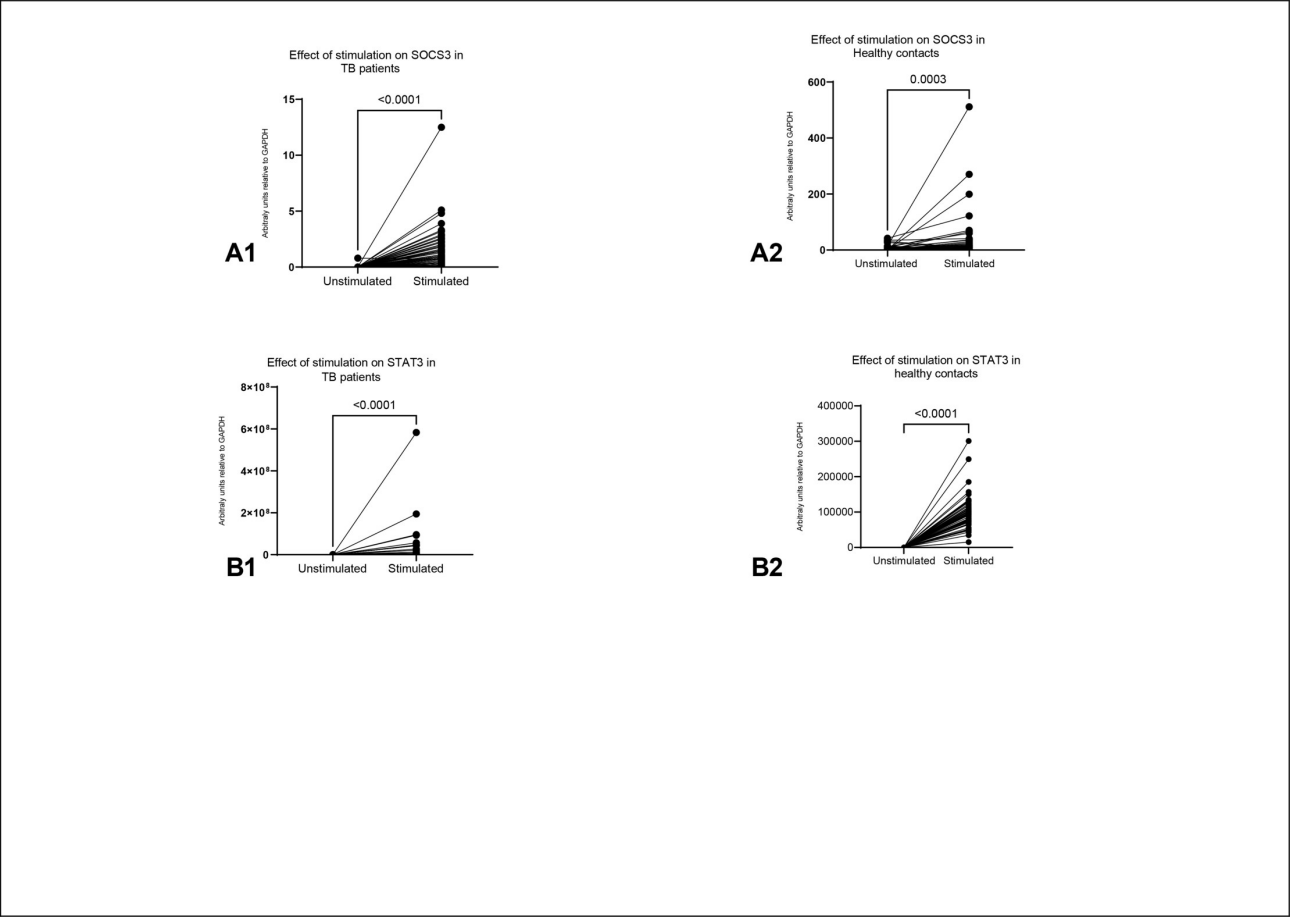
Shows the effect of stimulation with heat killed MTB for 24hrs (mimicking the response in expressing SOCS3 and STAT3 on exposure to MTB) on the expression of SOCS3 and STATS3 in the individual participant groups (TB patients and healthy contacts). A1: Shows that SOCS3 increased significantly post stimulation in TB patients, Median (IQR) 0.0 (0.0–0.0) in pre-stimulation to 0.7 (0.1–1.75), *p* value = <0.0001. A2: Shows significant rise in SOCS3 in healthy contacts post exposure to heat killed MTB, 1.4 (0.4–6.9) in pre-stimulation to 6.4 (1.65–21.95) post- stimulation, *p* value = 0.003. B1: Shows increased expression of STATS3 post stimulation in TB patients, 0.0 (0.0–0.0) in pre-stimulation to 408.6 (0.002–2,220,630) post-stimulation, *p* value = <0.0001. B2: Shows the shift in STAT3 expression following stimulation in healthy contacts, 0.1 (0.0–0.1) in pre-stimulation to 93,234 (6,684–118,833) post-stimulation, *p* value <0.0001. Statistical significance was evaluated using Wilcoxon signed-rank test.

Healthy contacts in comparison to the TB patients expressed significantly higher levels of inflammatory cytokines, IL-6, and IFNγ. In a like manner, we found that healthy contacts expressed higher levels of IL-10, an anti-inflammatory cytokine, than TB patients. Conversely, TB patients expressed significantly higher levels of TNF-***α*** than the healthy contacts. Further, we observed that only a few TB patients and healthy contacts expressed IL-2 and IL-17, hence a median of zero for both cytokines (Table 2). We observed a significant increase in the expression of SOCS3 and STAT3 in both cohorts post-stimulation with heat-killed mycobacteria tuberculosis (Fig 1).

**Table 2:** Expression of TH1 and TH2 cytokines in TB patients vs healthy contacts.

Variable	TB patients	Healthy contacts	*P* value
IL-10 (pg/mL)	58.72(9.67–197.3)	130.3 (43.6–227.1)	<0.0001*
IL-2 (pg/mL)	0.0 (0.0–59.02)	0.0 (0.0–81.75)	0.5765
IL-6 (pg/mL)	10,778 (5,540–19,711)	19,104 (11,252–35,267)	<0.0001*
IL-17 (pg/mL)	0.0 (0.0–1.73)	0.0 (0.0–60.01)	0.2941
IFNγ (pg/mL)	1.62 (0.0–20.57)	5.75 (0.59–24.66)	0.0570
TNF-α (pg/mL)	256 (70.88–1069)	140 (47.17–564.6)	0.044*

Intracellular cytokine profile in stimulated whole blood from 72 TB patients compared to 52 healthy contacts. The results are expressed as medians and interquartile range for interleukin (IL)-2, IL-6, IL-10, IL-17, interferon gamma (IFNγ) and tumour necrosis factor (TNF-**α**). Data expressed as medians (interquantile range) *represent statistically significant difference (P < 0·05) in a comparison of the expression of interleukins between TB patients and healthy contacts. Data.

**Fig 2:**
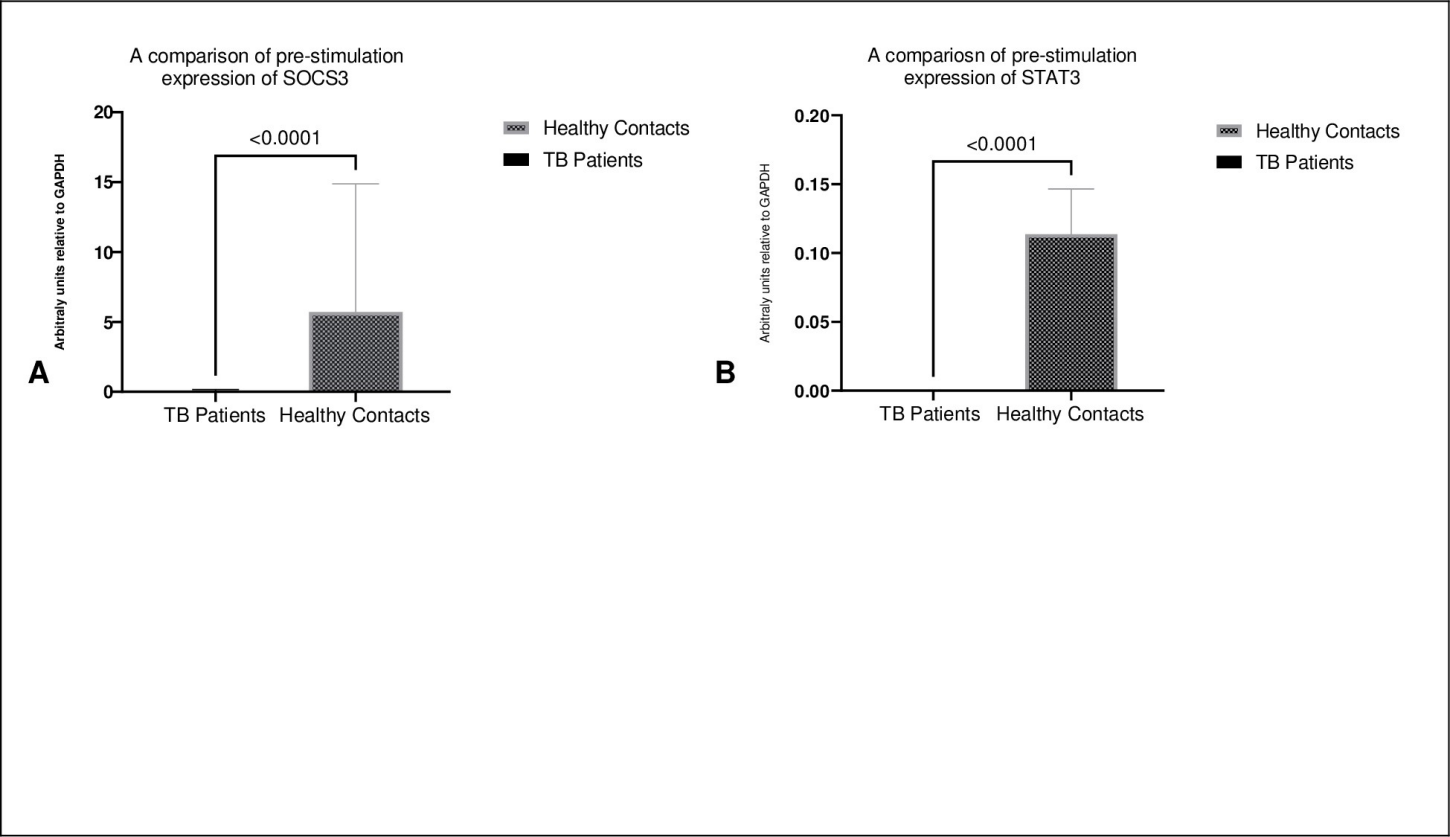
Shows the comparison of SOCS3 and STAT3 expression pre-stimulation in TB patients vs healthy contacts. A: Shows statistically significant higher levels of SOCS3 in healthy contacts when compared to TB patients. Median (IQR) 0.0 (0.0–0.0) in TB patients vs 1.4 (0.4–6.9) in healthy contacts, p value = < 0.0001. B. Shows the hardly any expression of STAT3 pre-stimulation in both TB patients and healthy contacts. Statistical significance evaluated using Mann–Whitney test.

Strikingly prior stimulation, TB patients narrowly expressed SOCS3 and STAT3. Contrariwise, healthy contacts expressed significant levels of SOCS3 prior to stimulation (Fig 2).

**Fig 3:**
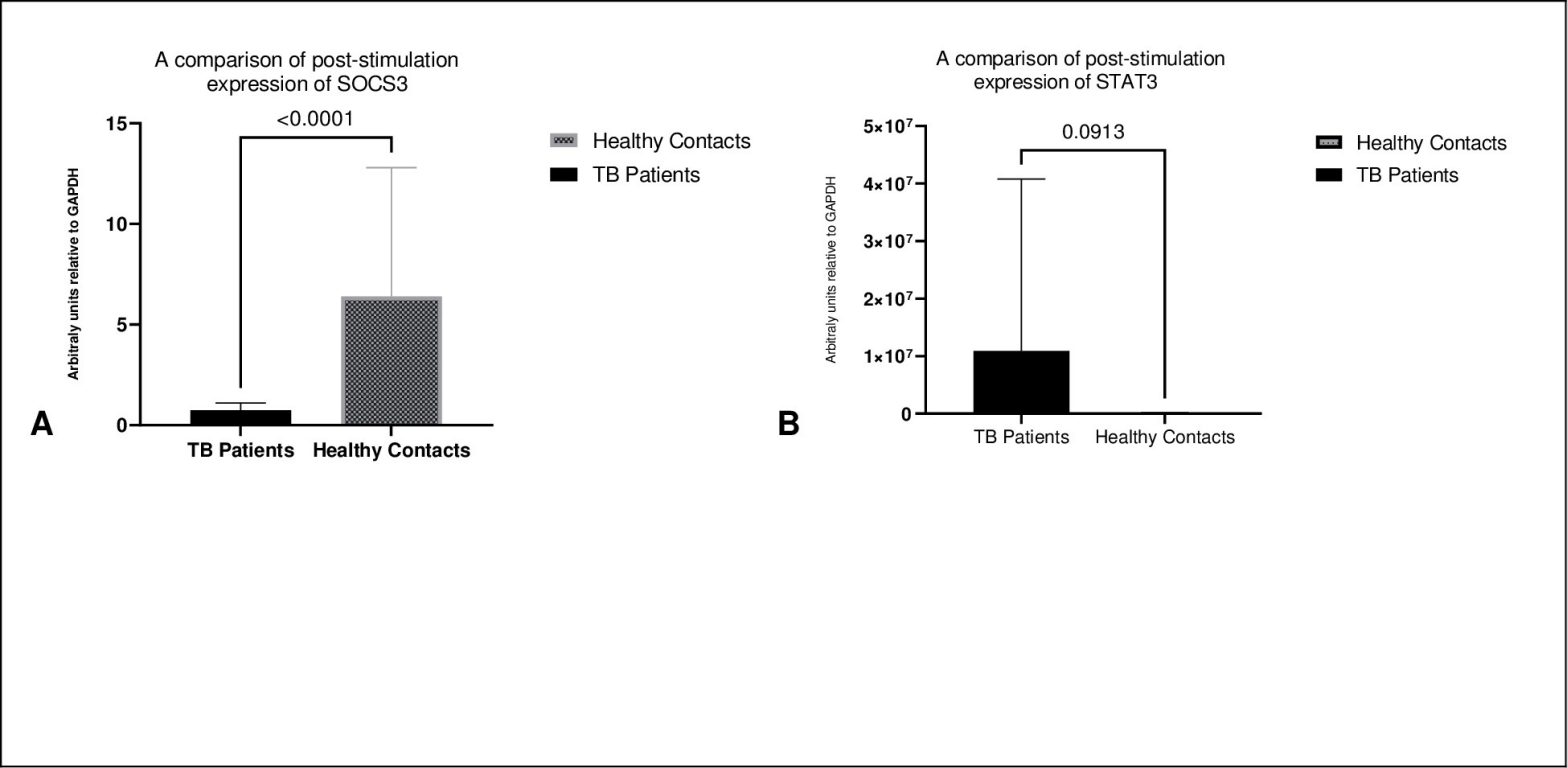
**A**: Shows significantly higher expression of SOCS3 in healthy contacts than TB patients post-stimulation. Median (IQR) 0.75 (0.175–1.8) in TB patients vs 6.4 (1.65–21.95) in healthy contacts, *p* value = < 0.0001. **B**: Shows no statistically difference in expression of in TB patients vs 93234 (966,847–118,833) in healthy contacts, *p* value = 0.0913. Statistical significance was evaluated using Mann–Whitney test.

A post-stimulation comparison of the expression of SOCS3 and STAT3 showed that healthy contacts expressed significantly higher levels of SOCS3 and on the contrary TB patients expressed higher levels of STAT3 (Fig 3).

**Fig 4:**
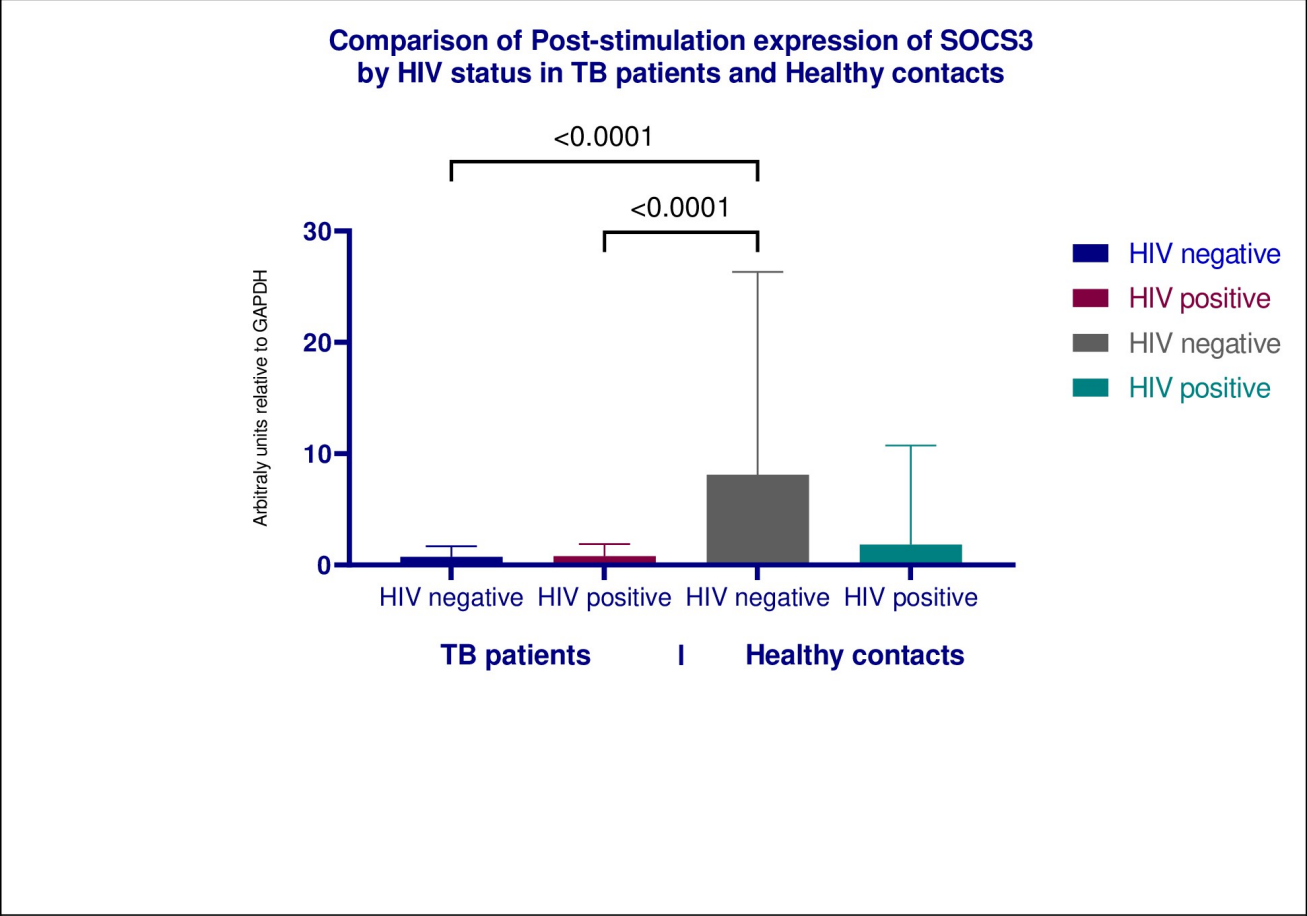
Shows multiple comparisons of post-stimulation expression of SOCS3 in TB patients and healthy contacts by HIV serostatus. HIV negative healthy contacts expressed significantly higher levels of SOCS3 in comparison to both HIV negative TB patients and TB/HIV patients, *P* values was < 0.0001 for individual evaluations. We found no statistically significant difference in the expression of SOCS3 between HIV positive healthy and HIV negative healthy contacts and both groups of TB patients.

**Fig 5:**
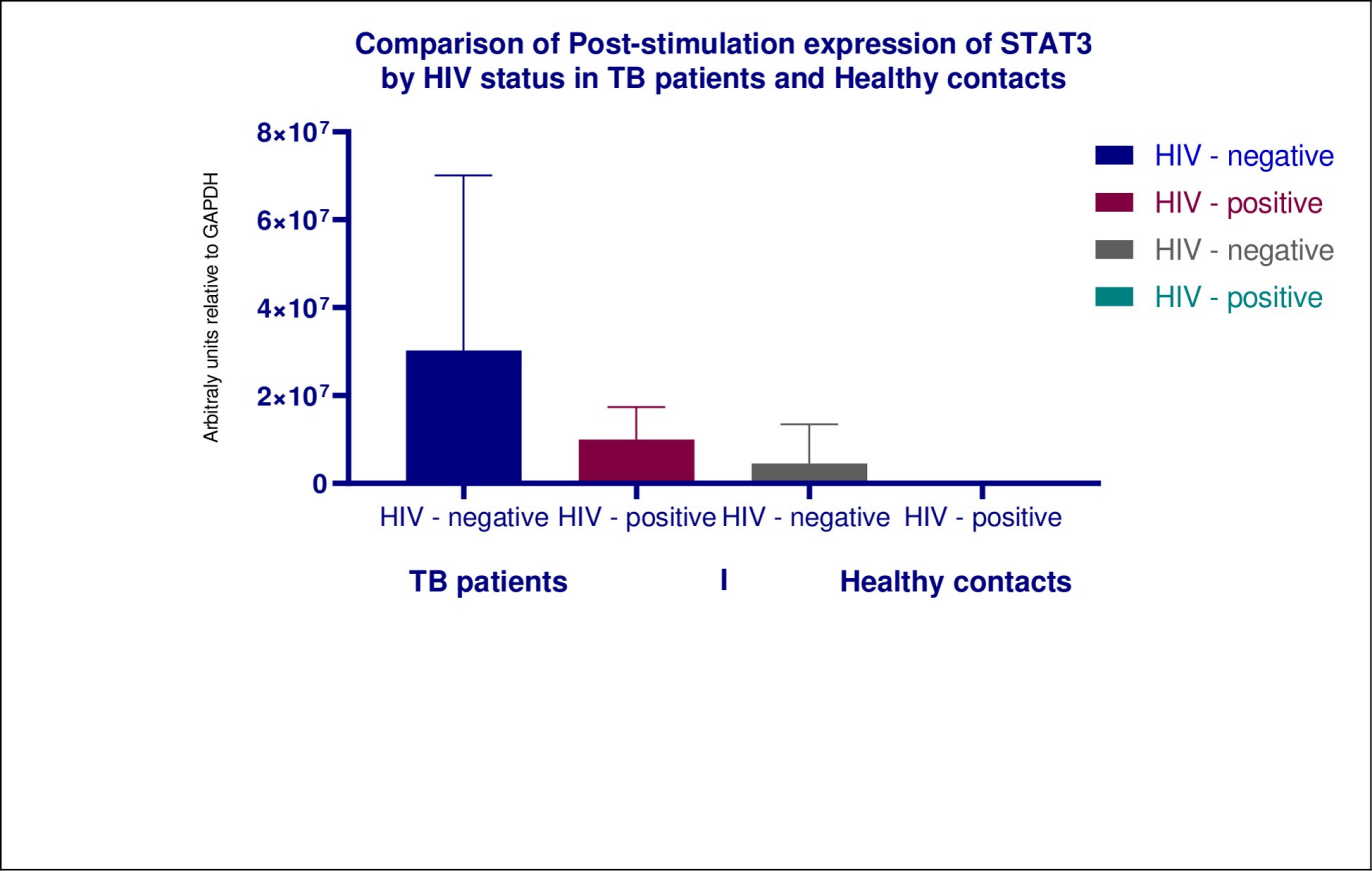
Shows column bar graph of the expression of STAT3 in TB patients vs healthy contacts stratified by HIV serostatus. We found no statistically significant difference in the expression of STAT3 between the categories of participants. Statistical significance was assessed using Kruskal–Wallis test.

We evaluated the effects of HIV infection on the expression of both SOCS3 and STAT3 in both populations. Firstly, we found no significant difference in expression of both SOCS3 and STAT3 within the cohort when stratified by HIV serostatus. Secondly, HIV negative healthy contacts significantly higher levels of SOCS3 than both groups of TB patients (Figs 4 and 5).

**Fig 6:**
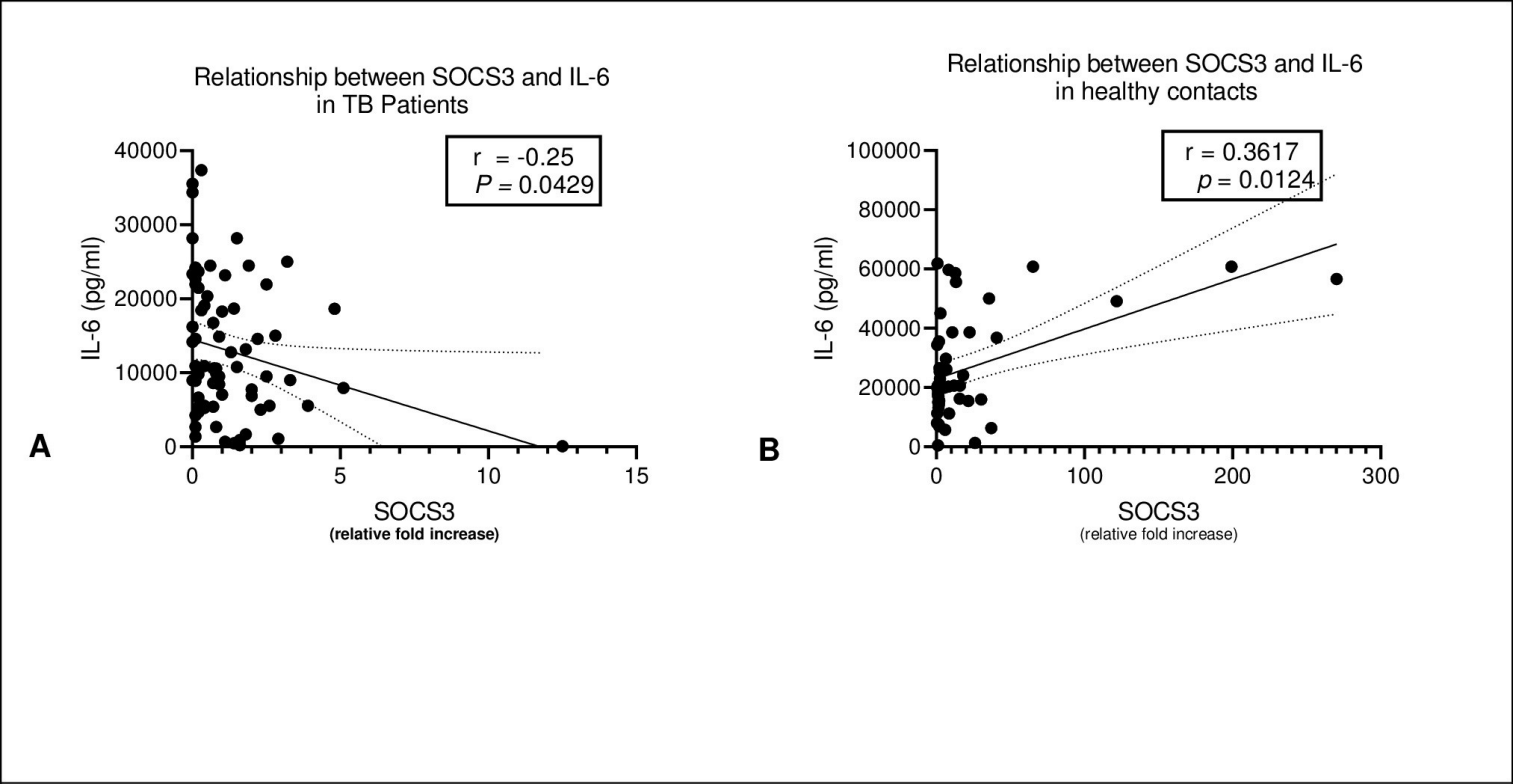
A: Shows a negative correlation between post-stimulation expression SOCS3 and IL-6 in TB patients and B shows a positive and significant correlation between post-stimulation expression SOCS3 and IL-6. The relationship between SOCS3 and IL-6 was assessed using spearman’s rank correlation.

To explore the relationship between SOCS3 and IL-6 in the both TB patients and healthy contacts, we conducted spearman’s rank correlation. In healthy contacts, we found that an increase in the expression of SOCS3 had a corresponding increase in IL-6. Conversely, in TB patients, we observed an inverse relationship between SOCS3 and IL-6 (Fig 6). Both observations were statistically significant.

## Discussion

Reduced expression of SOCS3 has been associated with increased susceptibility to intracellular pathogens TB inclusive and an increased likelihood of a lethal form of TB disease. Our study shows distinct expression of SOCS3 and STAT3 between TB patients and their healthy contacts. TB patients expressed disproportionately lower SOCS3 and higher STAT3 when compared to their healthy contacts. Both SOCS3 and STAT3 are central in mounting an effective immune response to mycobacterium tuberculosis. SOCS3 suppresses IL-6 activation of the Janus Kinase/STAT3, in turn, promotes an inflammatory response and preserves the expression of IFNγ [13].

In this study an increased expression of IL-6, IFNγ and IL-10 in healthy contacts in relation to TB patients. We think that the increased production of IL-6, IFNγ is influenced by the dominance of SOCS3. Healthy contacts demonstrate a balanced response between inflammatory and anti-inflammatory response. Evidence shows that a balance between pro and anti-inflammatory cytokines helps clear the mycobacterium tuberculosis in the macrophages and saves as a factor for containing the TB infection within the ghon focus [14,15].

Further, in this study, we note an interesting relationship between SOCS3 and IL-6 in the two cohorts. We found that there was a positive correlation between SOCS3 and IL-6 in healthy contacts and an inverse relationship between IL-6 and SOCS3 in TB patients. This suggests that SOCS3 promotes an inflammatory response. IL-6 a pleiotropic cytokine may either influence a pro or anti-inflammatory responses [16]. In animals, IL-6 has been established to be protective against TB, especially in preventing lethal form of TB [17]. The inverse relationship observed between IL-6 and SOCS3 in TB patients may be explained by the background effect of STAT3 which was dominantly expressed in TB patients [13]. Relating the findings on the relationship of SOCS3 and IL-6 to the outlined role of IL-6 in the pathogenesis of TB, we think low expression of SOCS3 leads to poor expression of IL-6. Therefore, low or lack of expression of SOCS3 increases susceptibility to TB.

As mentioned above we found a higher expression of STAT3 in TB patients when compared to healthy contacts. Our findings correlates with the findings of Queval and colleagues who established that STAT3 inhibits the production of nitric oxide a free radical that has anti-tuberculous properties and limits the proliferation of the mycobacterium tuberculosis bacteria in the granuloma [18]. This has negative effect on the response to exposure to TB leading to increased susceptibility to TB. This therefore, highlights the importance of the two genes in protective innate immunity to TB.

HIV disease is characterized by chronic inflammation. Miller et al in their study found higher expression of SOCS1/3 in the CD4 T cells which they thought inhibits JAK/STAT-S activation in HIV patients than the healthy controls [19]. They concluded that HIV interferes with SOCS-1/3 expression. Contrarily to the finding by Miller et al we did not note a statistically significant difference in the expression of both SOCS3 and STAT3 in with each cohort of participants when we stratified by HIV serostatus. Our plausible explanation for our observation firstly, is that all the HIV patients were on HAART. Secondly, is the difference in approaches, we used whole blood and Miller and colleagues used PBMCs. Cells perform differently when they are out of their milieu [20].

The clinical significance of these findings is that once optimised has potentials of being used to stratify the risk for TB disease based on the expression of SOCS3 and STAT3. This in turn may help in directing public health interventions such as TB preventive therapy in the at-risk population. We argue that SOCS3 can be targeted in stimulating protective immunity and in therapies for TB especially drug-resistant TB. Just to indicate the feasibility, Shi and colleagues demonstrated that targeting SOCS3 expression enhanced the phagocytic function of dendritic cells against candida [21]. Similar principles can be applied in TB therapy.

We did not explore the other family members of SOCS3 and STAT3, which may have had a background effect. It is therefore a limitation of this study. Though the mechanism responsible for determining which one between SOCS3 and STAT3 dominates remains un elucidated.

## Conclusion

Both SOCS3 and STAT3 are important genetic material in mounting protective innate immunity against TB. Our study demonstrates the relationship of the two genes and further shows that TB patients predominantly express STAT3. Given the function of the both STAT3 and SOCS3, dominance of STAT3 increases susceptibility to TB. SOCS3 influences the expression of IL-6 which is pivotal in mounting resistance to TB. We propose an exploration of SOCS3 stimulation and inhibition of STAT3 as possible approaches in gene therapy and vaccine development for TB.

## Supporting information

S1 File This is the S1 for the protocol used to quantify the expression of STATS3. (PDF)

S2 File This is the S2 for the protocol used to quantify the expression of SOCS3. (PDF)

S1 Data This is the S3 for Figs 1–6. (XLSX)
