# Supplementary material for: Expression of mycobacterium tuberculosis induced SOCS3 and STAT3 and the implications on innate immunity in TB patients vs healthy contacts in high TB/HIV endemic setting: A cross-sectional analytical study
Source: PLoS One. 2022 Jul 15;17(7):e0263624. doi: 10.1371/journal.pone.0263624 (PMC9286224; doi:10.1371/journal.pone.0263624)
Supplement: S2 File — (PDF) [file pone.0263624.s002.pdf]

Human SOCS3 qPCR primer pair

Catalog Number: **HP101201**

Shipping and Storage information

Catalog No.	Amount	Reaction number of 25-μl volume	Shipping and Storage	Package tube
HP101201	2 nmol	200	lyophilized powder	1

Information of the target gene and primers

Species	Gene Bank Ref.ID	Gene Symbol	PCR size (bp)	Other Name
Human	NM_003955.4	SOCS3	109	CIS3; SSI3; ATOD4; Cish3; SSI-3; SOCS-3

Validation Report

Positive tissues or cells：human U87 cell

The amplification curve and dissolution curve of gene SOCS3 in the qPCR experiment (cDNA and ddH_2O as templates).

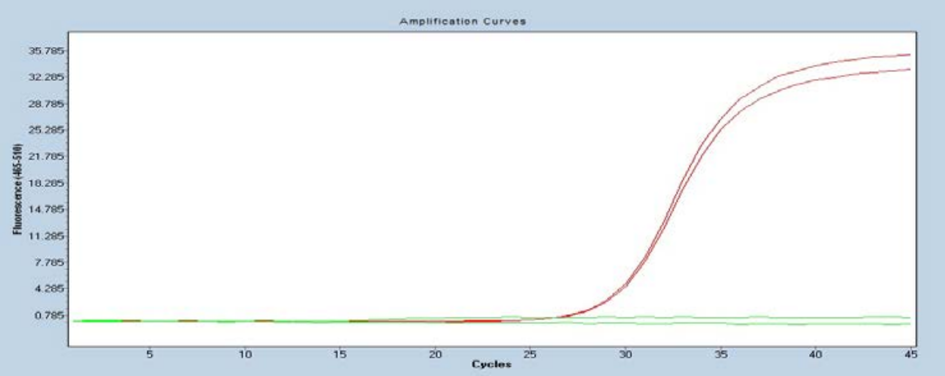

amplification curves

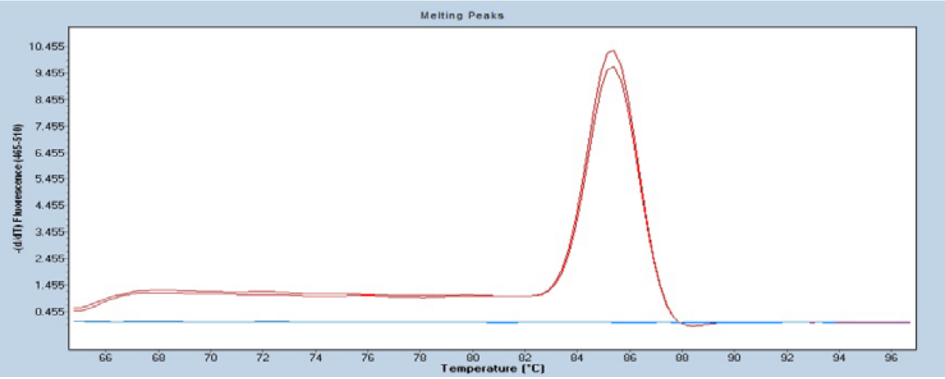

dissolution curves

Human SOCS3 qPCR primer pair

Catalog Number: **HP101201**

I Introduction of technology

qEASY qPCR Primer Pairs are designed using SBI's proprietary primer design algorithm. They are used for SYBR Green dye-based real-time PCR and designed according to the conserved region of all variants of a specific gene. At least one primer crosses the junction of adjacent exons to avoid amplification of genomic DNA directly and effectively. Our primer pairs cover all genes from human, mouse, rat and can be widely applied to the quantitative analysis of gene expression. cDNA used as templates, a single, correct-size band is produced in SYBR Green dye-based PCR with each pair of primers. Therefore, our primers have the characteristics with high specificity, high amplification efficiency, wide linear range and uniform reaction conditions. Each package for a specific gene is supplied with a lyophilized mixture of forward and reverse primers that can be used directly in SYBR Green dye-based real-time PCR after they are dissolved into ultrapure water.

II Product description

Shipping and Storage information

Shipping and Storage conditions: Each primer package contains a lyophilized mix of forward and reverse primers for a specific target gene in a centrifugal tube and is shipped at room temperature.

Amount	Reaction number of 25-μl volume	Shipping and Storage	Package tube
2 nmol	200	lyophilized powder	1

Upon receipt, store them at -20℃ and avoid repeated freeze-thaw cycles. When stored under these conditions and handled correctly, the product can be kept stably for 12 months in lyophilized powder and 6 months in solution.

Information of target genes and primers refer to page 1.

III Use process

The mix of forward and reverse primers of a specific gene is lyophilized powder, which is attached to the wall of centrifugal tube. Before using, centrifuge the tube for a few seconds, then re-suspend the primer mix in 200 μl dH_2O to make a final concentration of 10 μM. We recommend preserving them with small packages and storing them at -20℃. Avoid repeated freeze-thaw cycles.

The customer only needs to take into account the volume of PCR reaction system when using the primer pairs. Pipet a certain amount of dissolved primer mix to make a final concentration of 0.4μM and use it with SYBR Green qPCR Mix of other companies. The methods in detail refer to the **part IV "Experimental Procedures"**.

The product is suitable for detection by SYBR Green and not necessarily suitable for Taqman.

IV Experimental Procedures

For two-step RT-PCR protocols

1. Total RNA or messenger RNA (mRNA) must first be reverse transcriped into cDNA: the reaction mixtures contained total RNA (≤2 μg), 1μL OligdT(500 μg/mL), 1μL dNTP(10 nM) and Rnase-free ddH$_2$O in a final volume of 10 μL, mix gently and incubate for 5 min at 65 ℃, put on the ice 2 minutes immediately. Then add 2 μL 10×reaction buffer, 4 μL MgCl$_2$(25 mM), 2 μL DTT(0.1 M), 20 units of RNSEOUT, 200 units of superscriptⅡ RT enzyme and RNase-free ddH$_2$O in a final volume of 20 μL. Mix gently and incubate for 50 minutes at 42℃, then incubate for 5 minutes at 70℃.

2. The preparation of related reagents and materials for qPCR experiments: templates (first-strand cDNA or plasmid), qPCR Primer mix (10 μM), RNase-free H$_2$O, 2×SYBR Green PCR Master Mix. Keep the qPCR mix containing SYBR Green away from light, thaw it at room temperature (if stored at -20℃) and turn it upside down gently to mix, then place it on ice.

3. Prepare a master mix of PCR reagents with the components in the table below (e.g. SYBR Green I Master Mix of Roche).

Component	Volume/reaction (μL)	
2×SYBR Green mix (Roche)	10	25
qPCR Primer mix (10 μM)	0.8	2
Template (DNA or cDNA, ≤100ng)	5	5
ddH$_2$O	4.2	18
Total	20	50

4. Set up and run the program to carry out the PCR reaction according to the operating manual of machine(e.g. Light Cycler 480 Ⅱ). Generally three-step way is applied(annealing temperature is 60 ℃).

Step 1: pre-denaturation

95℃ 5min

Step 2: PCR reaction

95℃ 10s
60℃ 20s } 45 cycles
72℃ 10s

(Annealing temperature is alterable according to primers. The annealing temperature is 60℃ in our experiment)

Step 3: melting curve

95℃ 5s

65℃ 1min

65℃-97℃（continuous, ramp rate 0.11℃/s, acquisitions 5s/℃）

Step 4: 40℃ 30s

5. Analyze the data according to the machine's manual after the reaction is finished.

For one-step RT-PCR protocols

1. The preparation of related reagents and materials for qPCR experiments: template RNA; qPCR Primer mix (10 μM); RNase-free H_2O; 2×SYBR Green RT-PCR Master Mix; RT Enzyme mix. Keep the qPCR mix containing SYBR Green away from light, thaw it at room temperature (if stored at -20℃) and turn it upside down gently to mix, then lay it on ice.

2. Prepare a master mix of PCR reagents with the components in the table below (e.g. SYBR Green I Master Mix of Roche).

Component	Volume/reaction (μl)	
2×SYBR Green RT-PCR mix	10	25
qPCR Primer mix (10 μM)	0.8	2
RT Enzyme mix(10 U/μL)	0.2	0.5
Template (RNA, ≤100ng)	5	5
ddH_2O	4	17.5
Total	20	50

3. Set up and run the program to carry out the PCR reaction according to the operating manual of machine (e.g. Light Cycler 480 Ⅱ). Generally three-step way is applied (annealing temperature is 60 ℃).

Step 1: Reverse transcription

42℃ 10min

Step 1: Pre-degeneration

95℃ 30s

Step 2: PCR reaction

95℃ 10s
60℃ 20s } 45 cycles
72℃ 10s

(Annealing temperature is alteable according to primers. The annealing temperature is 60℃ in our experiment)

Step 3: melting curve

95℃ 5s

65℃ 1min

65℃-97℃（continuous, ramp rate 0.11℃/s, acquisitions 5s/℃）

Step 4: 40℃ 30s

4. Analyze the data according to the machine's manual after the reaction is finished.

V Verification process of qPCR primers and verification report

1. Validation Experiment Instrument: Roche Applied-science LightCycler®480 Ⅱ

2. Verification experiment materials and reagents

Reagents: Roche SYBR Green I Master mix, etc; qEASY qRCR primer mix
Materials: First-strand cDNA of different human tissues or cells

3. Validation Process

(1)The screening process of primers: design three pairs of primers in conserved regions for each specific gene. Trying them with the SYBR Green mix reagent by the qPCR experiment. The protocols in detail refer to the two-step RT-PCR protocol in the **part Ⅳ Experimental Procedures**.

(2) Generation of the verification report: through analyzing the amplification curves and dissolution curves of three pairs of primers, selected the pair of primers with high specificity and sensitivity.

4. Validation Report

The amplification curve and dissolution curve of a specific gene in the qPCR experiment (cDNA and ddH_2O as templates). The validation report refer to page 1.

VI Notes

1. In order to avoid genomic DNA amplification, at least one primer for specific gene crosses the junction of adjacent exons. However, it is necessary to treat total RNA with DNase I to remove genomic DNA for genes without introns.

2. The RNA with high quality and optimized reaction conditions are the prerequisites to ensure the accuracy of experiments. It is essential to operate carefully and avoid degradation during extraction of RNA. By adjusting the template concentration within the detectable linear scope and adding the positive control the experimental failure can be avoided .

3. Calculate the amplification efficiency of primers needs to draw standard curve generally. Prepare a dilution series (e.g., 5-fold or 10-fold dilutions) of a RNA or purified plasmid as templates to construct standard curves for primers by SYBR Green-based real-time RT-PCR. Construct a standard curve for the specific gene by plotting CT values (Y-axis) against the log of template amount or dilution (X-axis). Based on the formula $E = 10^{-1/slope}$ -1, the amplification efficiency E was calculated by the slope of standard curve (E lies between 90% to 105%).

4. Carry out the quantitative analysis based on the report provided by this product, please refer to the following methods:

(1) absolute quantification: The amount of the target gene is calculated by using the CT value of real-time PCR and the corresponding standard curve.

(2) relative quantification: If the amplification efficiencies of the target gene and the reference gene are close to 100%, less than 5%, the Livak method ($Ratio=2^{-\Delta\Delta CT}$) is suitable. If the amplification efficiencies of the target gene and the reference gene are not close, more than 5%, the Pfaffl method($Ratio=(E_{target})^{\Delta CT,\ target\ (calibrator-test)} / (E_{ref})^{\Delta CT,\ ref\ (calibrator - test)}$) is suitable.
